# A Response to the Toast “Dentistry”

**Published:** 1900-07

**Authors:** C. C. Barker

**Affiliations:** Meriden, Conn.


### A RESPONSE TO THE TOAST "DENTISTRY."[1]

BY DR. C. C. BARKER, MERIDEN, CONN.

I HAVE no desire, or even inclination, to claim anything for dentistry which does not rightfully belong to it.

I hold now, and always have held, that every vocation which has for its object true service in answer to the legitimate needs of humanity is honorable, and has claims of its own for proper consideration.

No argument is necessary to prove the need of dental service; and if the rank of dentistry among the numberless vocations is to be determined by the character of the service required, and the ability to render that service,—and, pray tell me, what other way to fix the dignity and grade of any calling can be worth mentioning but this,—then the proper practice of dentistry is no ordinary matter.

The character of dental service the world needs and requires cannot be fully stated within the compass of a few short sentences. (I am speaking only in outline to-night.)

If I were to speak upon this subject at length, I should dwell upon the purpose and priceless value of the dental organs. We have to set ourselves a-thinking to properly realize all this.

A person with a thoroughly sound set of teeth and a healthy mouth has an endowment—a working capital—of incalculable worth. Perfect teeth are a prime factor in good digestion; there

---

[1] Read at the fourth annual dinner of the Hartford Dental Society, March 31, 1900.

cannot be good health without good digestion, and without good health we cannot realize the full measure of our abilities as we attempt to solve the problems of life.

The perfect teeth are, however, very rare; the vast majority are victims of dental decay, and, unaided by dental service, are doomed to—— Well, it is not necessary that I present you dental gentlemen with a detailed list of the troubles which so often accompany and follow the lesion and breaking down of tooth tissues.

The first experience generally is that peculiar and trying pain which Bobbie Burns called "The hell o' a' diseases!"

And the curse of Caliban, who wished that "All the infections that the sun sucks up from bogs, fens, flats, on Prosper fall and make him by inch meal a disease!"

Caliban's curse seems only a mere trifle when we think of the abscesses, the necroses, the facial deformities, the neuralgias, the dyspepsias, and the various abnormal systemic conditions which can be traced to dental caries for their beginning. As one eminent writer has said, "Indigestion, largely induced by imperfect mastication, is the prevailing malady of civilized life, probably the occasion of more disorders than can be traced to any other cause."

The character of the service required is the arrest of decay and the preservation of the teeth for the exercise of their invaluable functions; or, if past remedy, their removal and artificial substitution. This is the mission and service of dentistry. And were we able to compass in our thought what this all means,—the prolonging of human life, the comfort and happiness of mankind,—we should conclude as never before that the mission of the dentist is really a beneficence and a blessing. But this is not all we are to think of; the teeth have other functions which should be remembered. "Without their assistance in vocalization, distinctness of utterance in either speech or song is impossible. And what other features combined can confer so much of beauty upon the human face as a fine set of teeth?" Nothing so surely mars manly beauty or female loveliness as a blight here. How delicately Tom Moore speaks of the lack:

> "What pity! blooming girl,
> That lips so ready for a lover
> Should not beneath their ruby casket cover
> One tooth of pearl!
> But, like a rose beside the church-yard stone,
> Be doom'd to blush o'er many a mouldering bone."

When we think of what dentistry may and does accomplish, we can also see that to this our chosen calling belongs the patent of true nobility.

This then—in brief statement—is the character of the service required and administered by capable practitioners of dentistry.

Now let us think of the ability requisite to properly render this service. Not every person can become a capable dentist. I do not know of any vocation which demands in its practitioners such varied and peculiar endowments—both natural and acquired—as does dentistry. Our esteemed colaborer, Dr. Crouse, of Chicago, editor of the *Dental Digest,* speaking of desirable qualifications in a dentist, said, "Is it too much to say that he needs all the logic of a lawyer, the scientific knowledge of the physician, and the high moral ideas and sense of responsibility of the clergyman combined?"

Dr. Crouse, as those of you who know him will say, is full of enthusiasm. Yes, and a reasonable amount of sensible enthusiasm is a good thing to have in any business. "Is it too much to say?" It is a large measure of ability, but it is not, after all, a full statement of requirement.

It must be confessed that people come to us not because they are altogether fond of our attention, but somewhat as a choice between two evils, and they are often in a very unwilling frame of mind. Here is where we have to apply the logic of the lawyer; not exactly in the mandatory sense, but by reasoning and persuasion. To do this well there must be a good knowledge of human nature, tact, and self-control.

How can a man take an instrument and safely and properly proceed to operate upon a person who has yielded confidence and placed himself under the dentist's hand, unless this dentist possesses an accurate knowledge of the tissues of the human system,—the bone, the muscle, arteries, capillaries, veins, nerves, the physiological functions of the various organs, their inter-relation, and the laws which govern vital action? And the dentist, as the physician, must know pathology, and be able to recognize the various expressions of disease. Therapeutics must be a part of his equipment, and his materia medica must be at hand.

The fact is, the practice of dentistry lies within the realm of medicine. This may be disputed and denied, but the fact remains, nevertheless.

There is another special endowment which is not really necessary to the general physician, which the surgeon needs in quite a measure, but without which the dentist can never hope to succeed; this is mechanical skill, and it must be possessed in high degree.

There is so much to be said along these lines, it is difficult to abridge. There must also be a knowledge of the materials employed, of metallurgy, and a good bit of chemistry. And withal, the dentist should be an artist.

When thus equipped, under what conditions does he perform his service? Under a stress of circumstance the like of which surround no other vocation. Within the very mouths of people which involuntarily resist intrusion, among tell-tale and protesting nerves, which scarce need to send telegrams the little way up to headquarters, for the two eyes are trained upon us all the while, and some of our patients possess very peculiar idiosyncrasies. But I will not dwell upon these features, except to say that dental operations must be performed under the stress of very adverse conditions, and well done, else they had better not be done at all.

There must be honor and integrity on the part of the operator; and to skilfully and faithfully perform these duties day after day and year after year, requires great powers of endurance.

If the rank of a vocation is to be fixed by the character of its service and the ability to render it, then there can be no doubt of the high professional status of dentistry.

It is "an occupation which involves a liberal education," and also that of a special sort.

Correct standards in dental practice are quickly recognized and sought after by broad-minded practitioners; and such men most naturally seek fellowship and association with others like themselves. It was this spirit, this touch of nature, this kinship, which prompted the formation of the first dental society; and it is the same spirit which finds illustration in this very pleasant and profitable gathering to-night.

Dental history, both written and unwritten, bears ample testimony that inscribed upon the honor-roll Hartford has had, and still has, a goodly number of bright and shining names. And Hartford seems determined to lead the van; for it must be said to your credit that your society is, so far as we are able to learn and to discover, the first to equip and maintain a permanent headquarters—a meeting-place—in one of the best of modern buildings, accessible always to every member, where the current literature of the profession is at hand, and opportunity for interchange of thought, free from the interruptions and routine of the practitioner's office.

Let it be remembered—never to be forgotten—that our associations—national, State, and local—have been, and still are, the conservators of all true progress.

It is their influence which has constantly been raising dentistry to higher levels, and has made of it what we see to-day. And this has been accomplished in spite of many discouragements and adverse tides. Every profession and vocation, so far as I know, is disgraced by a class possessed of moral obliquity who, while they wear the name, degrade and debase their calling, sometimes by sheer incapacity, or, what is quite as bad, perhaps worse, an abandonment of all principle, careless of all standards of equity, seeking only pelf and plunder. There is only one name that fits them, —I call them *pirates.*

What the future has in store for our profession I cannot exactly say. I am neither an optimist nor a pessimist. We are in a transition period; the adjustments are not altogether certain either way. But of this I feel sure, that when dangers threaten and storms beat and lower, the maintenance of correct standards by the sturdy, steadfast members of our societies throughout the land shall be our safeguard, our sheet-anchor; else shall dentistry, without chart, rudder, or compass, float and toss and drift hither and thither—a derelict, a tramp—on the wide, wild waste of waters, liable to both give and receive damage.

But whatever may come, men of Hartford, men of Connecticut, let us stand by our colors, remembering the truth of Charles Sumner's words that, "All defeats in a good cause are but resting-places on the road to victory at last."

